# Both gene expression for orotate phosphoribosyltransferase and its ratio to dihydropyrimidine dehydrogenase influence outcome following fluoropyrimidine-based chemotherapy for metastatic colorectal cancer

**DOI:** 10.1038/sj.bjc.6601335

**Published:** 2003-10-14

**Authors:** W Ichikawa, H Uetake, Y Shirota, H Yamada, T Takahashi, Z Nihei, K Sugihara, Y Sasaki, R Hirayama

**Affiliations:** 1 Second Department of Surgery, Saitama Medical School, 38, Moro-Hongo, Moroyama-cho, Iruma-gun, Saitama 350-0495, Japan; 2 Department of Digestive Surgery, Tokyo Medical and Dental University, Yushima, Bunkyo-ku, Tokyo 113-8519, Japan; 3 Department of Clinical Oncology, Saitama Medical School, 38, Moro-Hongo, Moroyama-cho, Iruma-gun, Saitama 350-0495, Japan

**Keywords:** orotate phosphoribosyl transferase, dihydropyrimidine dehydrogenase, fluoropyrimidine-based chemotherapy, metastatic colorectal cancer

## Abstract

Activation of 5-fluorouracil into its nucleotides requires phosphorylation by three pathways involving orotate phosphoribosyl-transferase (OPRT), uridine phosphorylase (UP), or thymidine phosphorylase (TP). In this study, we investigated the association between gene expressions of these three enzymes and antitumour effect. Gene expressions in primary colorectal tumours were analysed by a real-time reverse transcriptional–polymerase chain reaction method in 37 patients receiving oral treatment of tegafur-uracil and leucovorin for metastatic diseases. The median values of OPRT mRNA expressions were 1.39 and 0.85 for responding tumours and nonresponding tumours, respectively, showing a statistically significant difference (*P*=0.0008). Responding tumours had statistically lower expressions of TP mRNA than nonresponding tumours (*P*=0.006). However, there was no difference in UP mRNA expression between responding and nonresponding tumours. Patients with high OPRT (⩾1.0) gene expression survived longer than those with low OPRT (<1.0) expression. Dihydropyrimidine dehydrogenase (DPD) gene expressions were measured. Responding tumours had a statistically higher OPRT/DPD ratio than the nonresponding ones (*P*=0.003). When the median value of the OPRT/DPD ratio was selected as the cutoff value, patients with a high OPRT/DPD ratio survived statistically longer than those with a low ratio (*P*=0.0014). In conclusion, both the expression of OPRT gene and the OPRT/DPD ratio might be useful as predictive parameters for the efficacy of fluoropyrimidine-based chemotherapy for metastatic colorectal cancer.

An important anticancer agent widely used in the treatment of colorectal cancers, 5-fluorouracil (5-FU), is catabolised rapidly to the inactive metabolite dihydrofluorouracil (FUH_2_) by the first and rate-limiting enzyme-dihydropyrimidine dehydrogenase (DPD) (Heggie *et al*, 1987). The main mode of action of 5-FU is thought to be through its active metabolite: 5-fluoro-uridine-5′-triphosphate (FUTP) or 5-fluoro-2′-deoxyuridine-5′-monophosphate (FdUMP) (Danenberg, 1977). Metabolites such as FUTP can be incorporated into RNA, while FdUMP suppresses thymidylate synthase (TS), an essential DNA *de novo* synthetic enzyme that catalyses the methylation of deoxyuridine monophosphate (dUMP) to deoxythymidine monophosphate (dTMP) (Danenberg, 1977; Pinedo and Peters, 1988).

With combined DPD and TS gene expressions, both response and survival could be predicted more precisely than on the bases of only one gene expression. No tumour with both high DPD and high TS expression responded to tegafur-uracil (UFT), an oral fluoropyrimidine, and leucovorin (LV) therapy, but not even all tumours with both low DPD and low TS expression responded to the therapy, such cases having a response rate of 75% (Ichikawa *et al*, 2003). Salonga *et al* (2000) reported that only one of 12 tumours with both low DPD and low TS expression was a nonresponder to 5-FU, but that case had a high thymidine phosphorylase (TP) expression. These data suggested that combined evaluation of other gene expressions is needed to predict response more accurately.

While the importance of the value of DPD and TS for antitumour effects is recognised, the contribution of the different pathways to 5-FU activation is still unclear. Phosphorylation is necessary to activate 5-FU into its nucleotides by one or more of the following three pathways: (1) pathway 1: directly to FUMP by orotate phosphoribosyltransferase (OPRT) in the presence of 5-phosphoribosyl-1-pyrophosphate (PRPP); (2) pathway 2: indirectly to FUMP in a sequence of reactions with conversion of 5-FU to 5-fluorouridine (FUR) catabolised by uridine phosphorylase (UP) in the presence of ribose-1-phosphate (Rib-1-P); (3) pathway 3: indirectly to FdUMP by 2′-deoxy-5-fluorouridine (FUdR) catalysed by TP in the presence of deoxyribose-1-phosphate (dRib-1-P) (Figure 1

**Figure 1:**
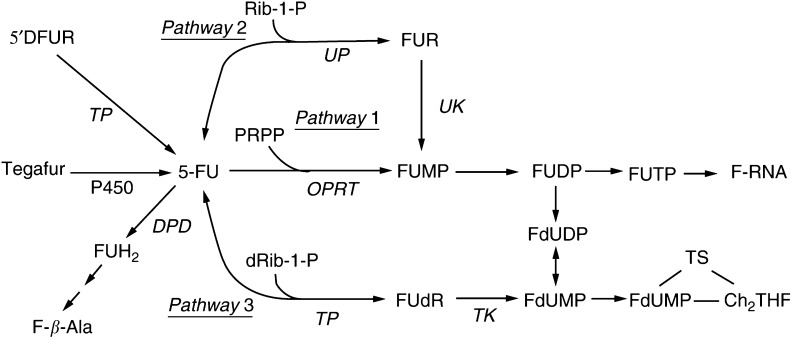
Metabolism of 5-FU, 5′DFUR, and Tegafur.

) (Peters *et al*, 1986,Peters *et al*, 1991).

The preferential use of the OPRT pathway (Pathway 1) was revealed to correlate with a higher sensitivity to 5-FU in cell lines and human xenograft models (Peters *et al*, 1986,Peters *et al*, 1991). Recently, in studies using human colorectal cancer tissues, a higher OPRT enzyme activity was observed in 5-FU-sensitive tissues than nonsensitive ones in *in vitro* chemosensitivity test (Isshi *et al*, 2002; Fujii *et al*, 2003). However, some investigators stated that the UP pathway (Pathway 2) is critical in the activation of 5-FU in experimental models (Schwartz *et al*, 1985; Cao *et al*, 2002). It is still controversial as to whether OPRT or UP gene expression is a key enzyme to activate 5-FU to predict the antitumour effect in the clinical setting.

Increasing TP activity is associated with the use of interferon (Evans *et al*, 1981), pyrimidine analogues (Schwartz *et al*, 1995; Ciccolini *et al*, 2000), or direct transfection of the TP gene (Evrard *et al*, 1999) to various cell lines and human xenograft models, which results in an increase in sensitivity to 5-FU. However, in the clinical setting, a high baseline level of gene expression of TP in colorectal tumours was associated with nonresponse in 5-FU/LV therapy (Metzger *et al*, 1998; Salonga *et al*, 2000). It is as yet unclear whether tumoral TP expression affects the antitumour effect of UFT/LV therapy on metastatic colorectal cancer.

In this study, we evaluated the predictive values of gene expressions of three phosphorylating enzymes, OPRT, UP, and TP, in UFT/LV treatment for metastatic colorectal cancer. In addition, the gene expression ratio of 5-FU-phosphorylating enzymes and the 5-FU degenerating enzyme DPD was also investigated for comparison with the antitumour effect, such as tumour shrinkage and survival.

## PATIENTS AND METHODS

### Clinical methods

The study population consisted of 37 patients with metastatic colorectal cancer, treated from July 1998 to December 2000 at the Department of Digestive Surgery, Tokyo Medical and Dental University, Tokyo, Japan. This study was approved by the Institutional Review Board of Tokyo Medical and Dental University, and all patients gave written informed consent.

These 37 cases represent all patients during that period who satisfied our enrollment criteria. They received the same treatment as first-line chemotherapy for metastatic disease, after resection of the primary tumour. Eligibility criteria, patient characteristics, and treatment regimens were previously described (Ichikawa *et al*, 2000,Ichikawa *et al*, 2003). Eligible patients had (1) a performance status score of 2 or better on the Zubrod scale (Eastern Cooperative Oncology [ECOG]) (Zubrod *et al*, 1960); (2) at least one measurable lesion; (3) adequate haematological, hepatic, and renal function; (4) life expectancy over 3 months. The median age was 62 years (range: 38–80 years). Nine patients had synchronous metastatic tumours at the time of resection of primary tumours and the other 28 patients had metachronous metastasis after resection of the primary tumours. Treatment consisted of 400 mg m^2^ day^−1^ oral UFT (in two doses q 12 h) and 15 mg body day^−1^ LV (in three doses q 8 h) for 5 days, followed by a 2-day rest period for four times during one 28-day cycle.

Before the treatment and after every two cycles (8 weeks) of treatment, measurable disease was reassessed by computed tomography. The response evaluation was based on standard UICC guidelines (Hayward *et al*, 1977). There were two complete responses (CR), 10 partial responses, 16 cases with no change, and nine cases of progressive disease, with a 32.4% response rate (95% confidence interval, 18.0–49.8%). All patients in this study are now off treatment; 36 of the 37 have died from cancer. One patient is still alive with CR at 23.0 months. The median follow-up time was 14.0 months.

Archival fresh frozen samples were obtained from the primary colorectal tumours at the time of surgery. No patient had received 5-FU chemotherapy preoperatively. Immediately after resection, the tumour sample was divided into two equal portions of at least 500 mg each, after removal of necrotic tissues. One portion was fresh frozen in liquid nitrogen until the time of RNA extraction and the other portion was embedded in paraffin to confirm histologically that it contained less than 5% contamination of normal tissue, necrotic tissue, and lymphocytes.

## LABORATORY METHODS

### Reverse transcriptional–polymerase chain reaction (RT–PCR)

Total RNA for each sample was isolated using the RNeasy mini kit (QIAGEN Inc., Chatsworth, CA, USA) according to the manufacturer's instructions (Chomczynski and Sacchi, 1987). For cDNA synthesis, reverse transcription using 10 *μ*g total RNA was performed in a total volume of 100 *μ*g containing 250 pmol oligo (dT)_18_, 80 U of RNasin (Promega, Madison, WI, USA), and 500 U Molony murine leukaemia virus reverse transcriptase (GIBCO BRL, Gaithersburg, MD, USA), 50 mM Tris-HCL (pH 8.3), 75 mM KCl, 3 mM MgCl_2_, 10 mM dithiothreitol, and 0.5 mM deoxynucleotide triphosphate (dNTP) solution.

### Real-time RT–PCR quantification

Quantitation of cDNAs of the genes of interest and an internal reference gene glyceraldehyde-3-phosphate dehydrogenase (GAPDH) was carried out using a fluorescence-based real-time detection method (ABI PRISM 7700 Sequence Detection System (Taqman); Perkin-Elmer Applied Biosystems, Foster City, CA, USA), as described previously (Gibson *et al*, 1996; Heid *et al*, 1996; Farrugia *et al*, 2003).

The PCR reaction mixture consisted of 600 nM of each primer, 200 nM probe, 2.5 U of AmpliTaq Gold Polymerase, 200 *μ*M each dATP, dCTP, dGTP, 400 *μ*M dUTP, 5.5 mM MgCl_2_, and 1 × Taqman Buffer A containing a reference dye, to a final volume of 25 *μ*l (all reagents Perkin-Elmer Applied Biosystems). Cycling conditions were 50°C for 10 s, 95°C for 10 min, followed by 46 cycles at 95°C for 15 s and 60°C for 1 min.

The primers and probe sequences used were as follows: OPRT primers: TCCTGGGCAGATCTAGTAAATGC and TGCTCCTCAGCCATTCTAACC, probe 6FAM (carboxyfluorescein)-5′-CTCCTTATTGCGGAAATGAGCTCCACC-3′TAMRA (*N*,*N*,*N*′,*N*′-tetramethyl-6-carboxyrhodamine); UP primers: TGACTGCCCAGGTAGAGACTATCC and AGACCTATCCCACCAGAAGTGC, probe 6FAM5′-TGCTCCAACGTCACTATCATCCGCAT-3′TAMRA; TP primers: CCTGCGGACGGAATCCT and GCTGTGATGAGTGGCAGGCT, probe 6FAM5′-CAGCCAGAGATGTGACAGCCACCGT-3′TAMRA; GAPDH primers: GAAGGTGAAGGTCGGAGTC and GAAGATGGTGATGGGATTTC, probe 6FAM5′-CAAGCTTCCCGTTCTCAGCC-3′TAMRA.

### Statistical analysis of RT–PCR results

TaqMan analyses yielded values expressed as the ratios between two absolute measurements (gene of interest/internal reference gene).

### Statistics

The Mann–Whitney *U*-test was used to compare the responders and nonresponders in terms of the related gene expressions. Spearman correlation was used to evaluate the association between the expressions of two genes. To evaluate the association with response, a two-sided Fisher's exact test was used. Survival was calculated from the onset of chemotherapy until death. One patient without any event was censored at the date of last follow-up. The overall survival curve was constructed using the Kaplan-Meier method, and differences were assessed by the log-rank test.

## RESULTS

The relative expressions of OPRT, TP, and UP mRNA were determined by real-time RT–PCR in 37 primary colorectal cancer specimens analysed previously for DPD gene expressions (Ichikawa *et al*, 2003). The median values of OPRT, TP, and UP mRNA expressions were 1.01 (range: 0.42–3.04), 70.0 (range: 10.0–249.8), and 2.13 (range: 0.56–6.30), respectively.

**Figure 2:**
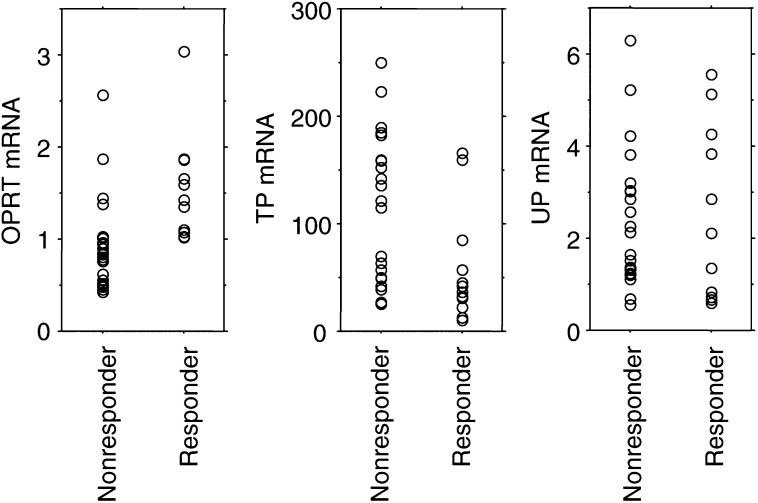
OPRT, TP, and UP gene expressions in terms of response. Responding tumours had a statistically higher OPRT gene expression or lower TP gene expressions than nonresponding tumours (*P*=0.0008 for OPRT and *P*=0.006 for TP). There was no difference in UP gene expression among responding and nonresponding tumours.

Tumours were categorised as either responding or not responding to a regimen of UFT and LV. The median values of OPRT mRNA expressions were 1.39 (range: 1.02–3.04) and 0.85 (range: 0.42–2.57) for responding tumours and nonresponding tumours, respectively, with the difference being statistically significant (*P*=0.0008, Mann–Whitney *U*-test) (Figure 2

). The median values of TP mRNA expressions were 39.3 (range: 10.0–165.6) and 121.6 (range: 25.5–249.8) for responding tumours and nonresponding tumours, respectively. Responding tumours had statistically lower expressions of TP mRNA than nonresponding tumours (*P*=0.006, Mann–Whitney *U*-test) (Figure 2). However, there was no difference between responding and nonresponding tumours in UP mRNA expression (median value, 1.73 (range: 0.59–5.56) for responding tumours *vs* 2.26 (range: 0.56–6.30) for nonresponding tumours) (*P*>0.05, Mann–Whitney *U*-test) (Figure 2).

The median values of 1.0 for OPRT and 70 for TP were selected for cutoff values separating high and low gene expressions of OPRT and TP. No responding tumours had low OPRT (<1.0) mRNA expression (Table 1

**Table 1:** Summary of response data for tumours with different expressions of OPRT, TP, and DPD genes

**Gene expression status**	**No. of responding patients**	**No. of nonresponding patients**	***P***
OPRT⩾1.0	12	7	
OPRT<1.0	0	18	<0.0001
			
TP⩾70	3	15	
TP<70	9	10	0.08
			
DPD⩾0.5	0	18	
DPD<0.5	12	7	<0.0001
			
DPD<0.5 and OPRT⩾1.0	12	1	
DPD⩾0.5 or OPRT<1.0	0	18	<0.0001

). A total of 19 tumours had high OPRT mRNA expressions of more than 1.0, with a corresponding response rate of 63% (12 out of 19) (Table 1). The response rates were 17% (three out of 18) and 47% (nine out of 19) in tumours with high TP (⩾70) expression and those with low TP (<70) expression, with a trend in favour of a lower TP mRNA expression for responding tumours (*P*=0.08, the two-sided Fisher's exact test) (Table 1).

**Figure 3:**
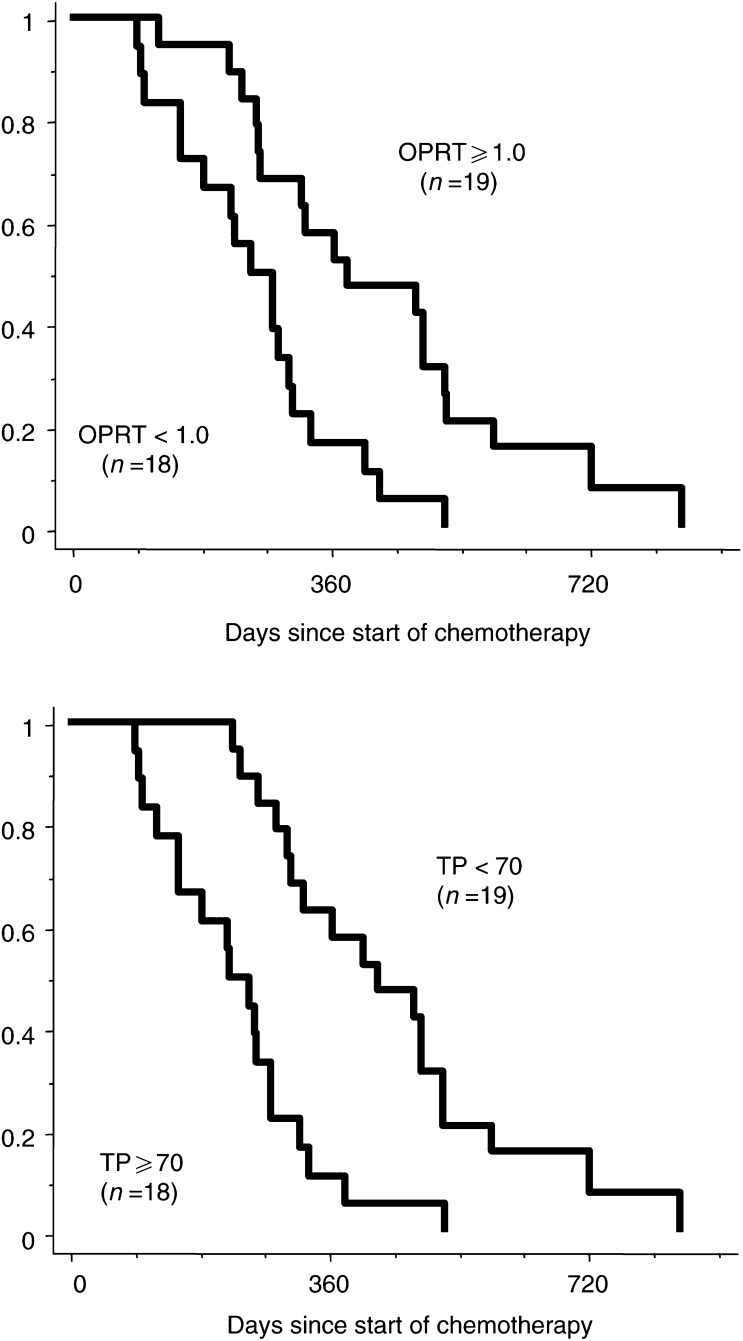
Correlation between prognosis and the expression of OPRT or TP.

The median survival was 12.5 months (range: 4.1–28.3 months) in patients with high OPRT expression, but 8.5 months (range: 3.0–17.3 months) in patients with low expression (*P*=0.0019; log-rank test) (Figure 3

). Patients with a low expression of TP mRNA survived longer than those with a high TP expression (median; 7.4 months ranging from 3.0 to 17.4 months for high expression *vs* 14.0 months ranging from 7.6 to 28.3 months for low expression, *P*=0.0002; log-rank test) (Figure 3).

**Figure 4:**
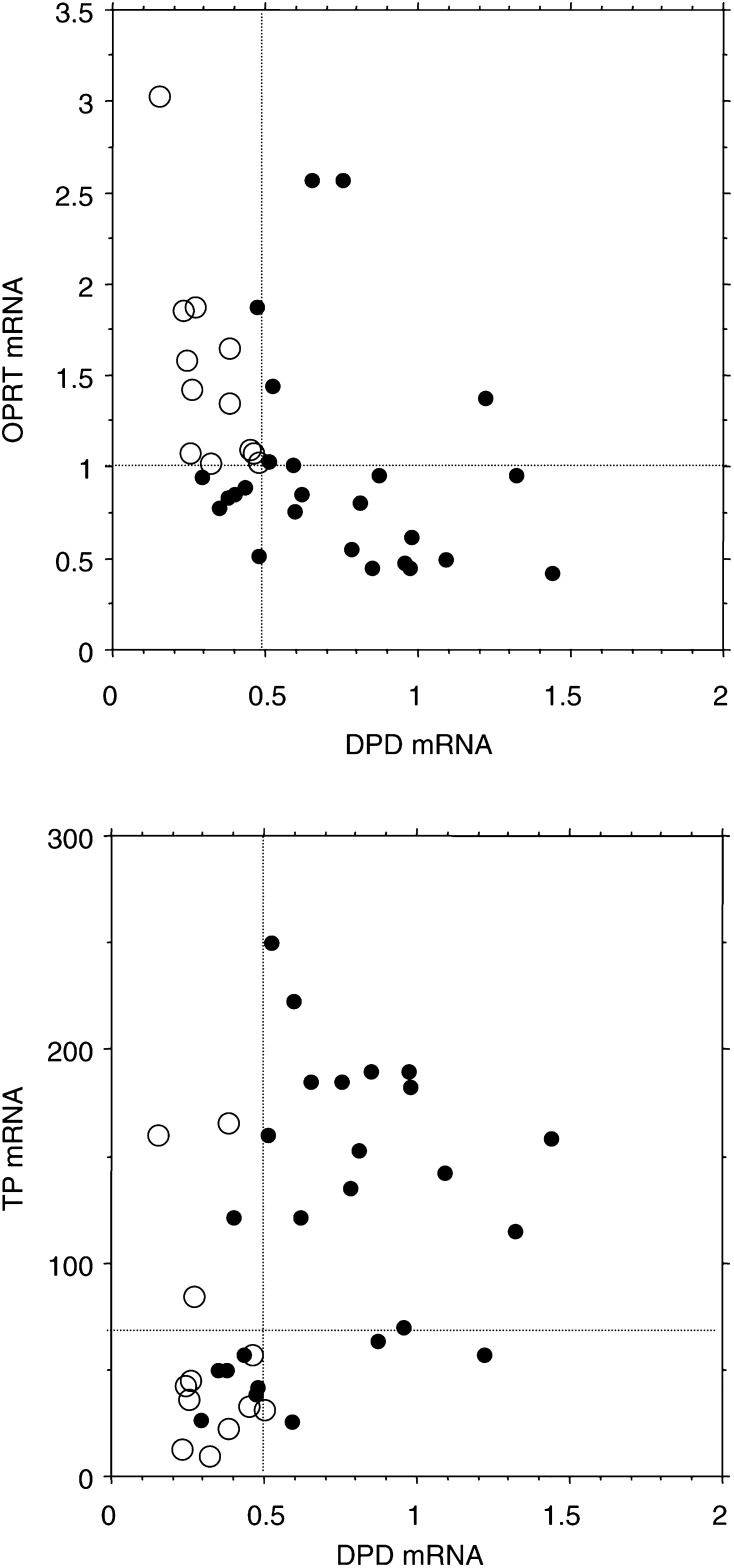
DPD expression plotted against OPRT or TP expressions. The values for DPD expression were obtained from a previously published study (Ichikawa *et al*, 2003). Solid and empty circles indicate nonresponders and responders, respectively. The dotted lines indicate the nonresponse cutoff values for each gene expression. Dihydropyrimidine dehydrogenase showed a negative correlation against OPRT expression (*r*=−0.529) and positive correlation against TP (*r*=0.520).

**Figure 5:**
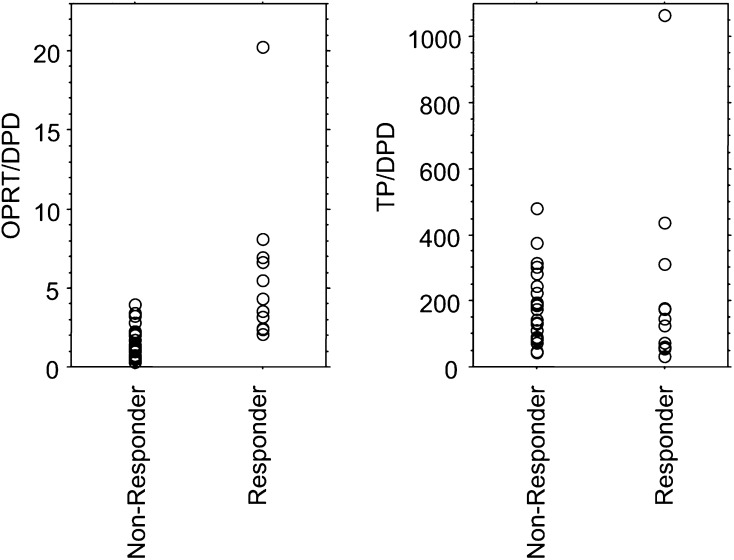
OPRT/DPD and TP/DPD ratios in terms of response. The responding tumours had statistically higher OPRT/DPD ratios than the nonresponding ones (*P*=0.003). However, the TP/DPD ratio had no statistical significance in terms of response.

**Figure 6:**
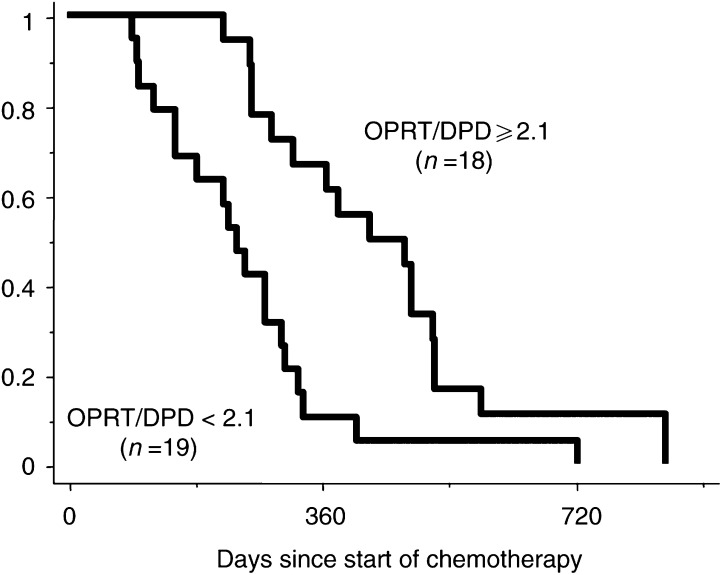
Correlation between prognosis and the expression of OPRT/DPD and TP/DPD.

Dihydropyrimidine dehydrogenase gene expressions were measured and the cutoff value for DPD was determined as 0.5, as previously reported in the same patient population (Ichikawa *et al*, 2003). A plot of DPD against OPRT mRNA expression showed a negative correlation between the expressions of these genes (Spearman rank correlation coefficient −0.529, *P*=0.0015) (Figure 4

). However, six of 19 tumours with low DPD (<0.5) expression had low OPRT expression, and six of 18 tumours with high DPD (⩾0.5) expression had high OPRT expression. In all, 12 of all 13 tumours with both low DPD and high OPRT responded (response rate: 92%), but no tumours with other combinations responded (Table 1). The responding tumours had a statistically higher OPRT/DPD ratio (median values; 4.3 (range: 2.1–20.2)) than nonresponding ones (median values; 1.3 (range: 0.3–4.0)) (*P*=0.003, Mann–Whitney *U*-test) (Figure 5

). When the median value of the OPRT/DPD ratio of 2.1 was selected as the cutoff value, patients with a high OPRT/DPD ratio (⩾2.1) survived statistically longer than those with a low ratio (<2.1) (median; 14.3 months ranging from 7.4 to 28.3 months for the high ratio *vs* 8.0 months ranging from 3.0 to 24.2 months for low expression, *P*=0.0014; log-rank test) (Figure 6

).

A plot of DPD against TP mRNA expression showed a positive correlation between the expressions of these genes (Spearman rank correlation coefficient 0.520, *P*=0.0018) (Figure 4). As previously reported, there were no responding tumours with high DPD expression. Among tumours with low DPD expression, the response rates were not statistically significant between tumours with a high TP (response rate: 75%) and those with a low TP (60%). The TP/DPD ratio had no statistical significance in terms of both response (Figure 5) and survival (data not shown).

## DISCUSSION

Our data demonstrate that there was a significant correlation between the OPRT expression level in tumour, and both the response and survival in patients treated with UFT and LV. An inverse correlation was observed between TP expression and the antitumour effect, and UP gene expression did not affect the tumour shrinkage and survival. In addition, the gene expression ratio of OPRT and DPD had a predictive value in the same patient cohort. This is apparently the first report to indicate a positive relationship between antitumour effect and OPRT expression in clinical materials.

*In vitro* and *in vivo* studies have shown that the main origin of TS-directed FdUMP metabolite comes from the reduction of FUDP by ribonucleotide reductase after prior conversion of FU to FUDP via OPRT, but not from FUdR phosphorylated by TP (Peters *et al*, 1986,Peters *et al*, 1991; Inaba *et al*, 1996; Koyama *et al*, 2000). In this study, TP negatively correlated with the antitumour effect in patients treated with a UFT and LV regimen, which agrees with previous reports on 5-FU and LV chemotherapy (Metzger *et al*, 1998; Salonga *et al*, 2000). Pathway 3 (phosphorylation of 5-FU to FdUMP by FUdR) is not important for anabolism of 5-FU because of the lack of the TP cofactor dRib-1-P at physiological concentrations (Barankiewicz and Henderson, 1977). Conversely, high TP concentrations in conjunction with the normally low dRib-1-P drive the reaction in the opposite direction (Ackland and Peters, 1999). These data support our findings that TP expression is a negative predictor of the result of treatment with the UFT and LV regimen.

Both TP and DPD are important enzymes in the pyrimidine salvage pathway (Fujiwaki *et al*, 2000). In addition, these two enzymes are associated with tumour aggressiveness. Thymidine phosphatase, which is identical to platelet-derived endothelial cell growth factor (Furukawa *et al*, 1992), and the degeneration products thymine and 2-deoxy-D-ribose have angiogenic (Ishikawa *et al*, 1989) and antiapoptotic effects (Kitazono *et al*, 1998). In human colorectal cancer, the DPD mRNA expression level was associated with a higher pathological classification, corresponding to the depth of invasion and lymph node metastasis (Shirota *et al*, 2002). The DPD gene expression of the liver metastasis was significantly higher than that of primary tumours. Based on these data, the moderately positive relationship between TP and DPD gene expressions in human colorectal cancer tissues is not surprising. Mori *et al* (2000) also reported that DPD protein expression strongly correlated with TP protein expression in colorectal cancer tissues, when measured by the enzyme-linked immunosorbent assay (ELISA) method (*r* 0.915, *P*<0.0001).

Recently, it has been reported that the TP/DPD ratio shows a significant correlation with the sensitivity of fluoropyrimidine analogues, such as 5′-deoxy-5-fluorouridine (5′-DFUR) and capecitabine (Ishikawa *et al*, 1998; Terashima *et al*, 2002). Ishikawa *et al* (1998) examined the relationships of tumour sensitivity to UFT with the TP/DPD ratio in xenograft models. There was no significant correlation between the TP/DPD ratio and tumour sensitivities to UFT. This report supports our result that the TP/DPD ratio was not related to the antitumour effect in terms of tumour shrinkage and survival in patients treated with the UFT and LV regimen. 5′-DFUR and capecitabine metabolise to 5-FU by TP and then the converted 5-FU is subsequently catabolised by DPD (Ishikawa *et al*, 1998). The TP/DPD ratio might be the predictor of antitumour effect only for 5′-DFUR and capecitabine, not UFT.

Generally, enzyme activities of both pyrimidine *de novo* synthesis and the salvage pathway in cancer tissues are upregulated more than those in normal tissues (Maehara *et al*, 1990; Peters *et al*, 1991). We also found a moderately negative correlation between DPD and OPRT gene expression. These data reflect Isshi's report that DPD enzyme activity correlates with OPRT enzyme activity in human colorectal cancer tissues (*r* −0.448, *P*<0.01) (Isshi *et al*, 2002). Orotate phosphoribosyl-transferase plays a role in the *de novo* pyrimidine synthesis pathway, which converts orotic acid to orotidine-5′-monophosphate (OMP) (Suchi *et al*, 1997). As mentioned above, DPD is categorised into the enzyme of the pyrimidine salvage pathway. Details of the underlying mechanisms remain unclear and further investigations are needed to clarify this molecular event.

If OPRT and DPD are coregulated, there would be little or no additional benefit for response prediction from measuring both enzyme expressions. The correlation between OPRT and DPD gene expressions was moderate (*r* −0.5299) and there were 12 cases (34%) that had low OPRT/low DPD or high OPRT/high DPD gene expressions. In all, 12 of 13 tumours with high OPRT and low DPD responded to UFT and LV therapy, having a response rate of 92%, but not all tumours with low OPRT or high DPD responded. Furthermore, the OPRT/DPD ratio was the predictor of antitumour effect to the UFT and LV regimen. UFT contains the 5-FU prodrug tegafur, which is converted by the P450 drug-metabolising enzyme in the liver (Kohne and Peters, 2000). The present study as well as recent reports (Isshi *et al*, 2002; Fujii *et al*, 2003) disclosed that the OPRT pathway was the most important to activate 5-FU among the three pathways. Hence, the ratio of OPRT and DPD, which is the rate-limiting enzyme of metabolism, was also evaluated. The OPRT/DPD ratios were statistically higher in responding tumours than in nonresponding ones and patients with high OPRT/DPD ratio tumours survived longer than those with low ratio tumours. Further studies are necessary to evaluate whether the OPRT/DPD ratio could be adopted as a possible predictor for the effectiveness of other fluoropyrimidine analogues.

In this study, UP gene expression had no predictive value for UFT/LV therapy. This result conflicts with previous reports of a positive relationship between UP expression and tumour sensitivity to 5-FU in mouse and rat cells (Schwartz *et al*, 1985; Cao *et al*, 2002). However, Cuq *et al* (2001) reported that UP overexpression did not increase the 5-FU sensitivity of human breast cancer cell. This discrepancy might be explained by species differences of UP expressions. Uridine phosphorylase increased in tumour tissues of mice and rats compared with human tissues, and the main enzyme of pyrimidine nucleoside phosphorylase is UP in rodents and TP in humans (Maehara *et al*, 1989).

Thymidylate synthase expression has the most evident value for the prediction of antitumour effect by fluoropyrimidine (Adlard *et al*, 2002). Previously, we reported that TS gene expression also had a predictive value to evaluate the antitumour effect in the same patient cohort (Ichikawa *et al*, 2003). As this study involved only a small number of patients, we could not evaluate the predictive value of TS when combined with DPD, OPRT, and TP. Large, prospectively randomised translational research studies are needed to apply predictive molecular markers in clinical practice, using standardised and validated assays.

We found that OPRT gene expression and the ratio of OPRT and DPD can be used to predict tumour shrinkage and survival in response to UFT and LV chemotherapy. Our study is a first step toward the goal of individualised cancer chemotherapy based on the fluoropyrimidine-related molecular characteristics of the tumour. However, the conclusions are drawn from a retrospective study consisting of a limited number of patients. Prospectively randomised translational treatment trials are needed to confirm our results and to define the best OPRT and OPRT/DPD cutoff points for each fluropyrimidine-based chemotherapy.
